# Identification of two transcription factors activating the expression of OsXIP in rice defence response

**DOI:** 10.1186/s12896-017-0344-7

**Published:** 2017-03-07

**Authors:** Yihua Zhan, Xiangyu Sun, Guozeng Rong, Chunxiao Hou, Yingying Huang, Dean Jiang, Xiaoyan Weng

**Affiliations:** 1 College of Life Science, Zhejiang University, Hangzhou, 310058 China; 2 Cixi Agricultural Technology Promotion Center, Cixi, 315300 China; 3 The Institute of Rural Development and Information Institute, Zhejiang Academy of Agricultural Sciences, Hangzhou, 310021 China

**Keywords:** 5’ deletion, OsbHLH59, OsERF71, Plant defence, Rice xylanase inhibitor, Transcriptional regulation

## Abstract

**Background:**

Xylanase inhibitors have been confirmed to be involved in plant defence. OsXIP is a XIP-type rice xylanase inhibitor, yet its transcriptional regulation remains unknown.

**Results:**

Herbivore infestation, wounding and methyl jasmonate (MeJA) treatment enhanced mRNA levels and protein levels of OsXIP. By analyzing different 5’ deletion mutants of *OsXIP* promoter exposed to rice brown planthopper *Nilaparvata lugens* stress, a 562 bp region (–1451 – −889) was finally identified as the key sequence for the herbivores stress response. Using yeast one-hybrid screening, coupled with chromatin immunoprecipitation analysis, a basic helix-loop-helix protein (OsbHLH59) and an APETALA2/ETHYLENE RESPONSE FACTOR (AP2/ERF) transcription factor OsERF71 directly binding to the 562 bp key sequence to activate the expression of *OsXIP* were identified, which is further supported by transient expression assay. Moreover, transcriptional analysis revealed that mechanical wounding and treatment with MeJA resulted in an obvious increase in transcript levels of *OsbHLH59* and *OsERF71* in root and shoot tissues.

**Conclusions:**

Our data shows that two proteins as direct transcriptional activators of *OsXIP* responding to stress were identified. These results reveal a coordinated regulatory mechanism of OsXIP, which may probably be involved in defence responses via a JA-mediated signaling pathway.

**Electronic supplementary material:**

The online version of this article (doi:10.1186/s12896-017-0344-7) contains supplementary material, which is available to authorized users.

## Background

Xylanase inhibitors (XIs) are a kind of plant-produced proteinaceous inhibitor that inhibits the activity of xylanase [1]. Recently, RIXI, riceXIP, OsXIP and OsHI-XIP xylanase inhibitors have been identified in rice plants [2–5], which all belong to XIP-type XIs. XIs have been thought to be involved in plant defence mainly for the reason that XIs only inhibit xylanases of microbial origin but not of plant origin. And many data provide evidence that XIs do indeed participate in plant defense [1].

Xylanase inhibitor genes act as defence-responsive genes in stress-induced signal transduction pathways. *Taxi-Ia* expression was induced 2.5 times and the transcripts of *Taxi-Ib/III* and *Taxi-IIb/IV* rose up to 20-fold by *F. graminearum* infection of wheat lemma, palea and ovary [6]. Infestation of wheat leaves by the powdery mildew fungus *B. graminis* induced the expression of *Taxi-Ib/III* and *Taxi-IIb/IV* [6]. The transcripts of *OsXIP* and *riceXIP* were drastically induced by wounding and methyl jasmonate (MeJA) treatment in the root [2]. Our previous study also revealed that pathogens can induce the expression of the rice xylanase inhibitor gene *RIXI* [7]. In planta direct evidence for this role has not been reported until Moscetti et al. [8] found that constitutive expression of the xylanase inhibitor TAXI-III delayed *Fusarium* head blight symptoms. Furthermore, overexpression of the RIXI xylanase inhibitor improved disease resistance of rice to the fungal pathogen, *Magnaporthe oryzae* [9]. In addition, overexpression of *OsHI-XIP* enhanced resistance in rice to herbivores, which is also the first time that a xylanase inhibitor has been demonstrated to play a role in resistance among rice herbivores [5]. However, the molecular basis underlying the regulation of XIs in plant defense is poorly understood.

A number of biotic and abiotic stress-responsive elements were observed by comparative analysis of cis-elements of xylanase inhibitors gene promoter by bioinformatics softwares PLACE and PlantCARE. The promoter region of a gene can provide valuable information about the factors inducing expression. For instance, cis-acting elements implicated in pathogen- and wound-inducible gene expression, i.e., GCC-box and W-box sequences could be recognized in the promoter region of *TAXI-III* [6]. Also investigation of the durum wheat *Xip-II* upstream region revealed the presence of a number of cis-acting elements controlling the expression of defense-related genes such as several W-boxes and a Myb-binding element, supporting its role in plant defense against pathogens [10]. The importance of these promoters regions has not yet been confirmed by promoter deletion analyses.

OsXIP is a XIP-type rice xylanase inhibitor, which was induced by various stresses such as MeJA treatment and wounding. And the expression patterns of OsXIP and riceXIP resemble each other and the induction of their expression by wounding may occur via a JA-mediated signaling pathway [2]. However, whether OsXIP plays an important role in resistance to invaders via a JA-mediated signaling pathway remains unclear.

Despite all these observations, there have been no reports on in planta functional characterization of the promoter region of xylanase inhibitor gene and its transcriptional regulation pattern so far. In this study, the promoter of *OsXIP* was cloned and analyzed, and a 562 bp region (−1451 to −889) was identified as the key sequence for the herbivores stress response by promoter deletion analyses. Using this 562 bp sequence as the bait, OsbHLH59 [11] and OsERF71 [12] proteins as direct transcriptional regulators of *OsXIP* responding to stress were identified. Collectively, our results, for the first time, reveal a transcriptional regulatory mechanism of OsXIP involved in defence responses.

## Methods

### Plant materials, growth conditions and stress treatments

The rice genotypes used in this study were Nipponbare wild-type (WT) and transgenic lines (see below). Rice seeds were sown in water and grown in normal culture solution in a greenhouse with natural day length extended to light/dark cycle of 14/10 h using high-pressure sodium lamp, with heating or ventilation used to maintain temperature at 28 °C and 18 °C during day and night respectively.

For wounding stress, 14-day-old seedlings were cut into 5–10 mm width and floated on distilled water. For phytohormone treatment, 14-day-old rice seedlings were submerged in 200 μM MeJA solution for 0, 2, 6, 12 and 24 h, and then shoots and roots were harvested separately. For BPH treatment, plants were individually infested with 20 adult BPH confined in a glass cylinder [diameter 4 cm, height 8 cm, with 48 small holes (diameter 0.8 mm)], the top of which was covered with a piece of sponge. One empty cylinder was used for control plants (non-infested).

### Construction of the *OsXIP* promoter vectors and rice transformation

The full-length *OsXIP* gene promoter named OP1 (−2070 bp to +52 bp) was amplified from the genomic DNA of WT with OP1-U (forward primer) and OP1-L (reverse primer). Then a series of nested 5’ deletions of OP1 fragments OP2 (−1451 bp to +52 bp), OP3 (−889 bp to +52 bp), OP4 (−569 bp to +52 bp), OP5 (−380 bp to +52 bp), OP6 (−172 bp to +52 bp), OP7 (−90 bp to +52 bp) were amplified by PCR from pMD19T-OP1 using the common reverse primer OP1-L and either the forward primers OP2-U, OP3-U, OP4-U, OP5-U, OP6-U, or OP7-U, respectively. The primers are shown in Additional file 1: Table S1. The full-length promoter and 5’-deletion derivatives were cloned into the pBI101.3-GUS upstream of *GUS* (β-glucuronidase). Empty vector pBI101.3-GUS was used as a negative control (VC). All constructs were mobilized into *Agrobacterium tumefaciens* EHA105 and transformed into calli derived from mature seeds of rice according to a previously described protocol [13, 14]. Approximately 90 calli were co-cultured for each vector, with the number of putative independent transformed plants being regenerated being 25, 19, 20, 16, 22, 13, 17 and 10 respectively for OP1-OP7 and a vector control. Primary transformants (T0) were raised, transferred to soil and allowed to grow in a greenhouse. Seeds were harvested and used for analysis in the next generation.

### RNA extraction and quantitative RT-PCR

Total RNA was isolated from roots and shoots of rice seedlings using the RNAprep pure plant kit (Tiangen) according to the manufacturer’s protocol. RNA (1 μg) was used to synthesize the first strand complementary DNA (cDNA) with an oligo (dT) primer according to the instruction of the PrimeScript first-strand cDNA synthesis kit (Takara). The qRT-PCR assay was performed on LightCycler480 instrument (Roche) using a SYBR® Premix Ex TaqTM kit (Takara). A rice actin gene *Osactin* (GenBank: Os03g50885) was used as an internal standard to normalize cDNA concentrations. The primers for qRT-PCR are listed in Additional file 2: Table S2. The relative quantification of gene expression was analyzed by the comparative method (2^−ΔΔCt^) [15] with some modifications. Using the 2^−ΔΔCt^ method, data were presented as the fold-change in mRNA expression normalized to the endogenous reference gene (*Osactin*) and relative to the control.

### Western blot analysis

OsXIP-specific polyclonal antibody was produced against a 15-residue synthetic peptide sequence of OsXIP (CGGRRNGVYRPFGDA) by GenScript USA Inc (China). Anti-OsXIP rabbit polyclonal antibody or anti-β-actin mouse monoclonal antibody (Beijing ComWin Biotech Co.,Ltd, CW0096) was used as the primary antibody. Immunoblot analysis was performed as described by Akulinkina et al. [16]. Samples were prepared from leaves of 2-weeks old rice plants treated with BPH for 24 h, wounding for 12 h and MeJA for 12 h, respectively. Protein fractions were separated by SDS-PAGE, and then transferred onto nitrocellulose membrane. Finally, immune complexes on a membrane were detected with BCIP/NBT. The reaction was stopped after 3 minutes of incubation by rinsing the membrane with water.

### Quantitative GUS analysis

Quantitative GUS activity was measured according to the method described by Jefferson et al. [17] with some modifications. Briefly, the shoots or roots of 14-days seedlings that carried different fragments of OP1 were homogenized in GUS extraction buffer (50 mM PBS, pH 7.0, 10 mM EDTA, pH 8.0, 20% methanol, 0.1% Triton X-100, 0.1% sodium lauryl sarcosine, and 10 mM β-mercaptethanol). Crude protein extract (50 μl) was added to 450 μl of extraction buffer containing 2 mM 4-methylumbelliferyl-β-D-glucuronide (MUG) at 37 °C for 30 min or 60 min, and thereafter 200 μl of the reaction mixture was added to 800 μl of stop buffer (0.2 M Na_2_CO_3_). The 4-methylumbelliferone fluorescence was measured using a spectrofluorophotometer (RF-5301PC, Shimadzu) at 460 nm with excitation at 355 nm. Protein concentration was quantified by methods described by Bradford [18]. GUS activity was calculated as pmol of 4-methylumbelliferon (4-MU) min per minute and per milligram of total soluble proteins and presented as GUS activity relative to the VC.

### Yeast one-hybrid (Y1H) screening

The Y1H screening used the Matchmaker Gold One-Hybrid Library Screening System (Clontech, Cat. Nos. 630491). The bait sequence (562 bp fragment) was cloned into the pAbAi vector that harbors the *AUR1-C* gene, conferring resistance to Aureobasidin A (AbA, a cyclic depsipeptide antibiotic used as a yeast selection marker). The resulting pAbAi-Bait construct was then linearized and integrated into the genome of the Y1HGold yeast strain by homologous recombination to generate a bait-specific reporter strain. The minimal inhibitory concentration of Aureobasidin A for the bait-specific reporter strain was determined. And the strain was used to screen a cDNA library generated from the leaves of WT treated by BPH for 24 h. The transformants were initially screened on selective medium (SD/−Leu/AbA^100^) and the positive colonies were identified by PCR and DNA sequencing.

For the re-transformation assay, the full-length CDSs of candidate genes were amplified from cDNA using the primers 59-AD-U/L and 71-AD-U/L as listed in Additional file 1: Table S1. The PCR products were then cloned into the pGADT7 vector and the resulting constructs were transferred into the bait reporter yeast strain mentioned above, respectively. The cells were grown on SD/−Leu and (SD/−Leu/AbA^100^) plates at 30 °C for 3 days, and resuspended in liquid media to OD_600_ of 0.1 (10^−1^) and diluted in a 10× dilution series (10^−2^ to 10^−3^). Of each dilution, 7 μl was spotted on media selecting for both plasmids (SD/−Leu) and selecting for interaction (SD/−Leu/AbA^100^), supplemented with 100 ng ml^−1^ to suppress background growth. The empty vector pGADT7 was used as a negative control.

### Chromatin immunoprecipitation (ChIP)-PCR analysis

The 35Sp::OsbHLH59:GFP and 35Sp::OsERF71:GFP expression vectors were constructed by subcloning the full-length CDSs without terminators of OsbHLH59 and OsERF71 into the pCAMBIA1300-sGFP vector under the control of the 35S promoter [19], respectively. The primers 59-GFP-U/L and 71-GFP-U/L are listed in Additional file 1: Table S1. The resulting constructs were then introduced into *Agrobacterium tumefaciens* strain EHA105, and transformed into WT rice.

2–3 g of 3–weeks-old 35S:OsbHLH59-GFP or 35S:OsERF71-GFP transgenic seedlings were used for ChIP-PCR experiments as described in Haring et al. [20]. In brief, transgenic rice was fixed with 60 mL of 1.0% formaldehyde by vacuuming for 10 min. The chromatin DNA was sheared to 200–500 bp fragments by sonicating. Sheared DNA was incubated with GFP antibody (Biogot) (ChIP). Chromatin before immunoprecipitation was used as an input control. The primers for PCR of the target DNA are listed in Additional file 3: Table S3.

### Transient expression in *Nicotiana benthamiana* leaves

The construction of vectors and transient expression in *Nicotiana benthamiana* leaves were performed as described by Ding et al. [21]. Briefly, for construction of effector vectors, the full length ORFs of OsbHLH59 and OsERF71 were amplified and cloned into the pCAMBIA1300-sGFP vector under the control of the 35S promoter. The 5’–deleted OP2 and OP3 promoters were constructed into the reporter vector pGreenII0800-LUC [22]. The recombinant plasmids were transferred into the *Agrobacterium* EHA105 lines. Then the EHA105 lines were co-infiltrated into the *N. benthamiana* leaves as described previously [23]. The Firefly and Renilla luciferase activities were quantified using a Dual Luciferase assay kit (Promega, http://www.promega.com/).

### Subcellular localization

The 35Sp::OsbHLH59:GFP and 35Sp::OsERF71:GFP expression vectors were infiltrated into the *N. benthamiana* leaves by *Agrobacterium*-mediated transformation [24]. The two constructs were also transfected into rice protoplasts according to the protocol of Yoo et al. [25] with some modifications. The rice protoplasts were isolated from stems of 12-day-old WT seedlings by enzyme hydrolysis. Then 10 μg plasmid DNA was polyethylene glycol/calcium-transfected into these protoplasts. Empty vector was used as a control. The cells were observed with a confocal microscope (Zeiss LSM 710).

## Results

### Herbivore infestation, wounding and methyl jasmonate (MeJA) treatment enhanced mRNA levels and protein levels of OsXIP

**Fig. 1:**
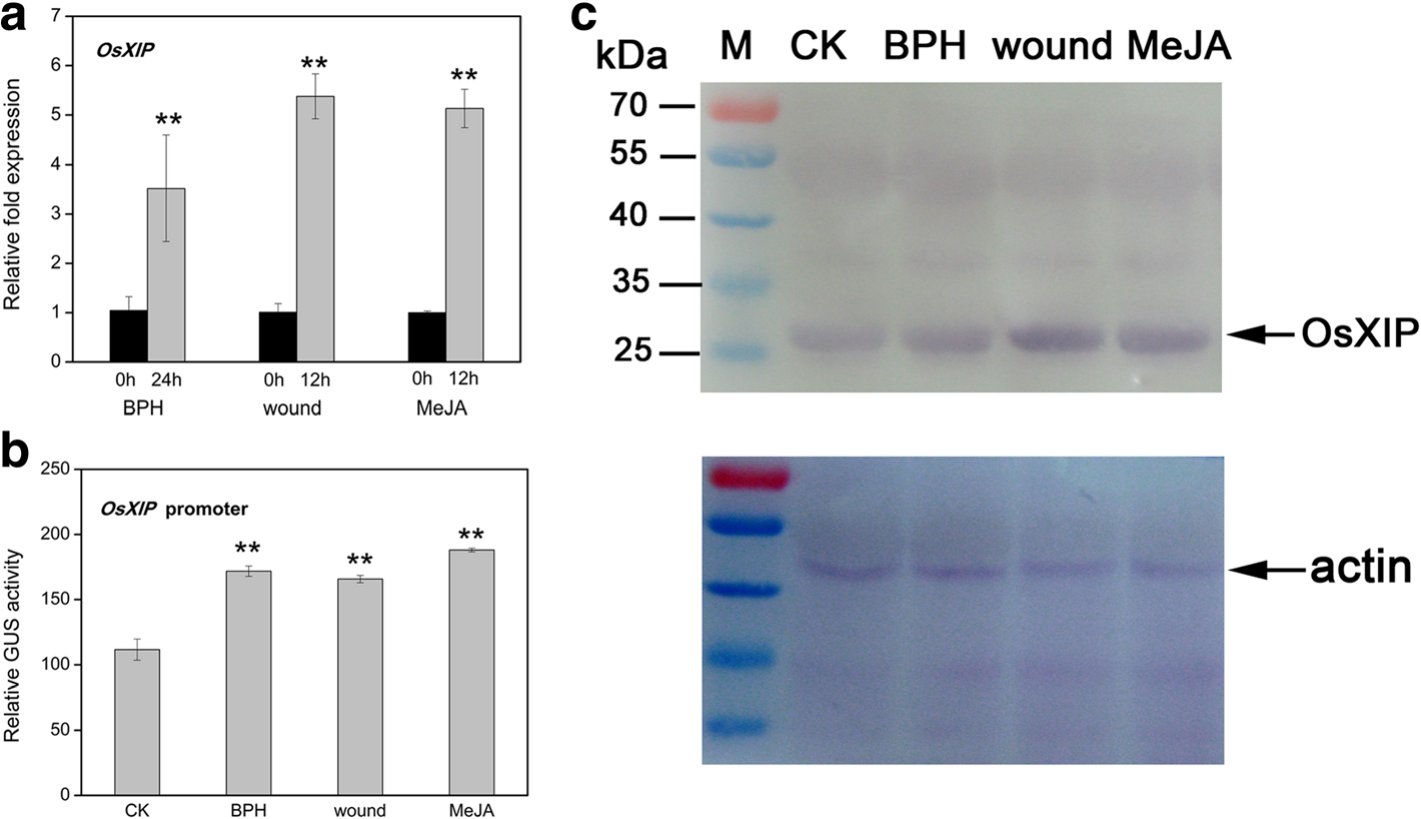
Inducible expression of OsXIP after different treatments. **a** Transcript levels of *OsXIP* in rice leaves after different treatments were analyzed by qRT-PCR. Two-weeks-old WT seedlings were treated with BPH for 24 h, wounding for 12 h and MeJA for 12 h, respectively. The data represent means ± SD of three independent replicates. Asterisks indicate statistically significant differences compared with CK (0 h) (**P < 0.01; Student’s *t* test). **b** GUS activity of OP1 transgenic rice after different treatments was measured by quantitative fluorescence method. Two-weeks-old transgenic rice seedlings were treated with BPH for 24 h, wounding for 12 h and MeJA for 12 h, respectively. The data represent means ± SD of three independent replicates. Asterisks indicate statistically significant differences compared with CK (**P < 0.01; Student’s *t* test). **c** Western blot analysis of OsXIP. Protein fractions were isolated from leaves of WT treated with BPH for 24 h, wounding for 12 h and MeJA for 12 h, respectively and subjected to immunoblot with anti-OsXIP antibody (top panel). M, markers of proteins, the sizes of the markers are indicated at the left of the picture. Fractions corresponding to 10 μg were loaded into each lane and equal loading was confirmed by anti-actin antibody (bottom panel)

qRT-PCR analysis revealed herbivore infestation, mechanical wounding, and MeJA treatment, especially wounding, resulted in an obvious increase in transcript levels of *OsXIP* (Fig. 1a). *OsXIP* expression was induced 3.5 times by BPH infestation and five-fold by wounding and MeJA treatment. To determine whether these stress also affected the protein levels of OsXIP, we analyzed the protein levels of OsXIP by quantitative GUS activity of transgenic rice that carried the full-length *OsXIP* gene promoter named OP1 (−2070 bp to +52 bp) and western blotting. The expression levels of GUS all significantly rose up (Fig. 1b). Immunoblot analysis showed that the level of OsXIP also increased upon these stress (Fig. 1c). These results demonstrate that *OsXIP* is a stress-responsive gene.

### Identification of herbivore-responsive promoter region

**Fig. 2:**
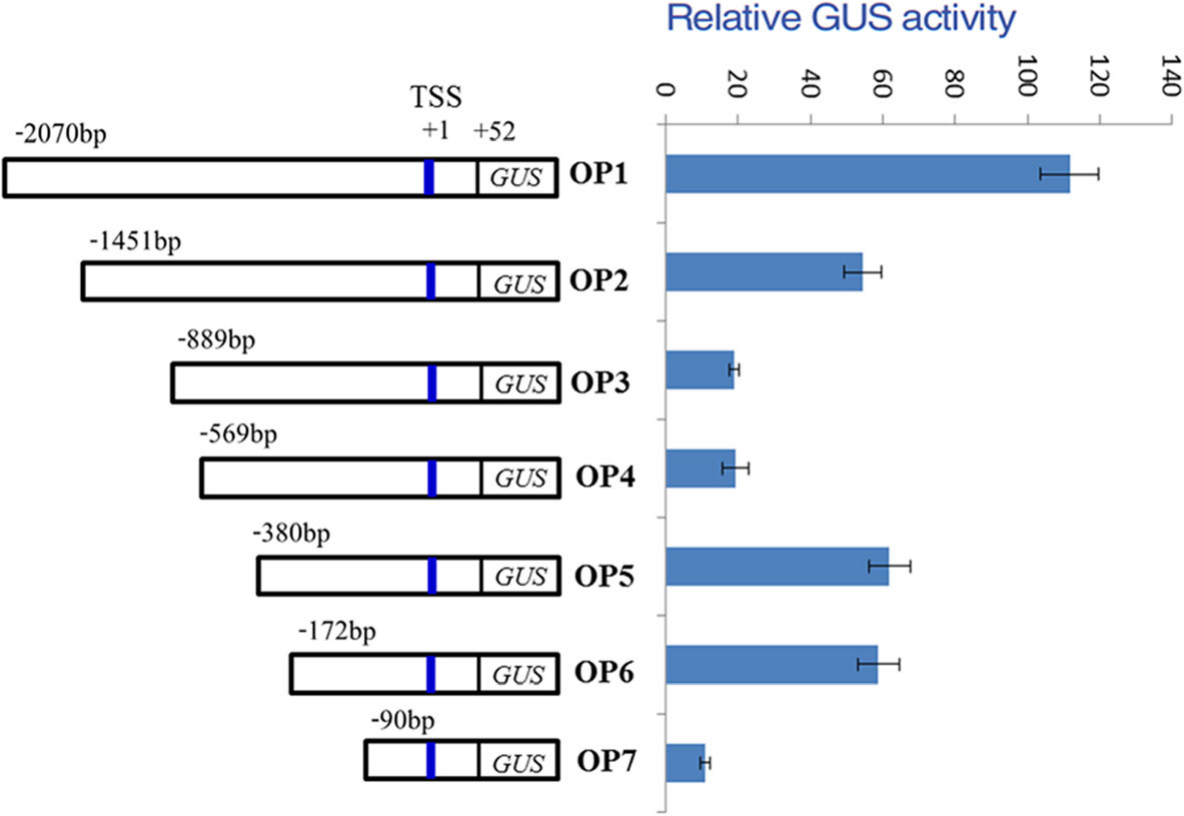
Schematic representation of 5’ deletion promoter constructs and their GUS expression in shoot tissues. TSS, transcriptional start site. For every construct, three independent T2 transgenic lines were measured, and similar results were obtained. The data represent means ± SD of three independent replicates

Given the observation that the expression of OsXIP was induced by different stress (Fig. 1), we used the PLACE and PlantCARE to analyse the promoter sequence of *OsXIP*. And as expected, a series of biotic and abiotic stress-responsive cis-regulatory elements exist in the promoter region (Additional file 4: Figure S1), such as W-box (TGACY) element, ABRE (CACGTG) element, MYB-binding (CGGTCA) site. To further determine the key sequences of the *OsXIP* promoter responding to herbivory, the transgenic plants that carried a set of 5’ deletion promoter reporter constructs, OP2, OP3, OP4, OP5, OP6 or OP7 were obtained (Fig. 2) and their quantitative GUS activity was measured.

Without BPH treatment (Fig. 2), about 110- and 54-fold higher expression of GUS was detected with OP1 and OP2 in the shoots, respectively, compared with vector control (VC). It was interesting to observe a sudden drop in expression with OP3, which was 18-fold higher compared with VC and similar to OP4. Surprisingly, the relative GUS expression for OP5 and OP6 are both three-fold greater than OP3 and OP4, despite having a greater deletion in the promoter region. Both OP5 and OP6 retain restore the expression level observed in OP2, whereas OP7 with the greatest promoter deletion has very minimal expression.

**Fig. 3:**
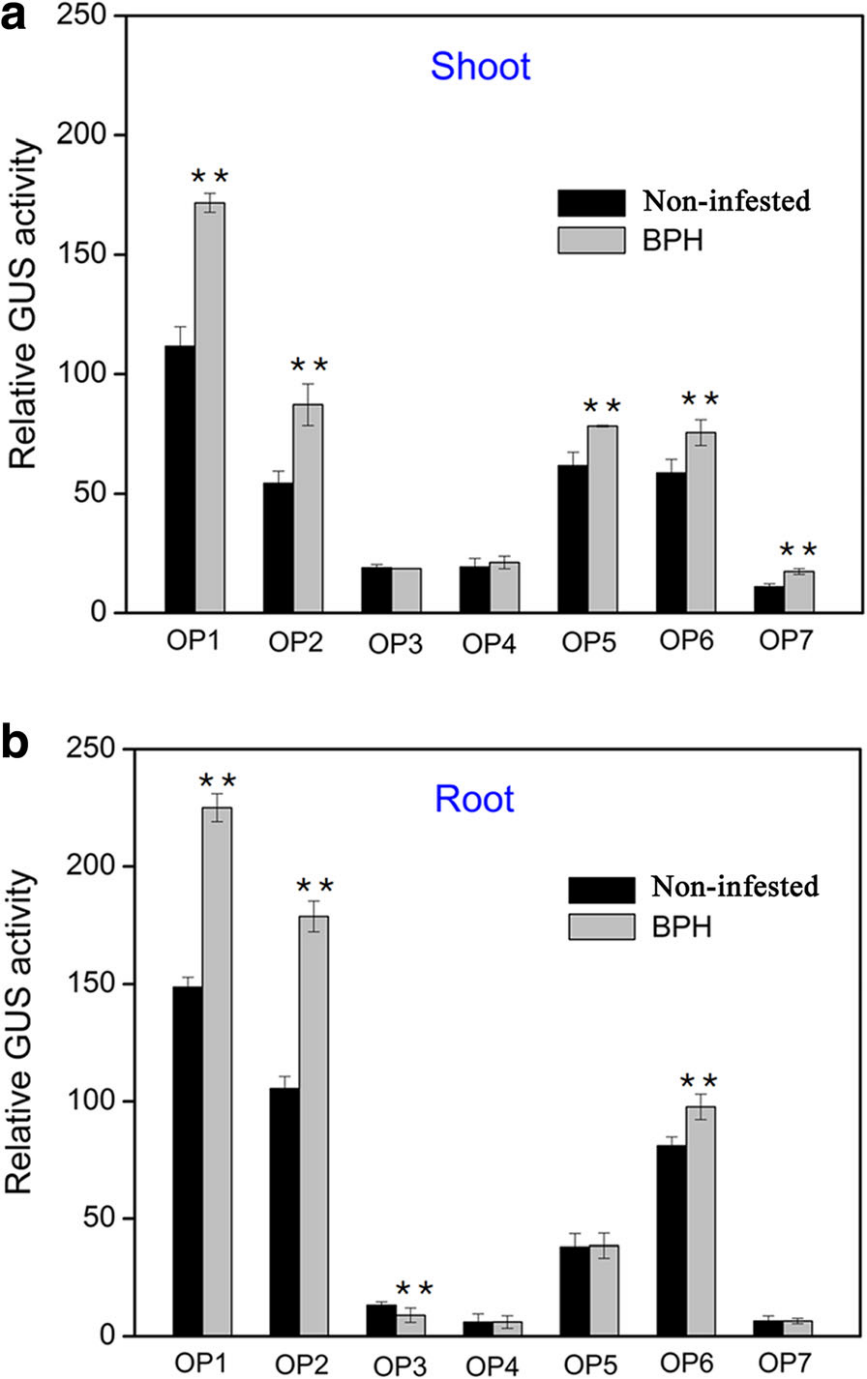
GUS expression of 5’ deletion promoter constructs infested by BPH in T2 transgenic plants. Relative (to VC) GUS activity in shoots (**a**) and roots (**b**) of different transgenic lines (OP1–OP7) infested by BPH for 24 h. For every construct, three independent T2 transgenic lines were measured, and similar results were obtained. The data represent means ± SD of three independent replicates. Asterisks indicate statistically significant differences compared with control (Non-infested) (**P < 0.01; Student’s *t* test)

After infestation with BPH for 24 h (Fig. 3), GUS expression increased significantly in both the shoots and roots of transgenic lines OP1 and OP2. Elevated expression of GUS was also observed in the shoots of OP5, OP6 and OP7 and root of OP6. However, no significant change in expression was observed in the shoots of OP3 and OP4 and roots of OP4, OP5 and OP7. A sudden decrease in GUS expression was observed in the root of OP3. As mentioned above, the GUS expression of OP1 and OP2 was induced after BPH infestation, but OP3 and OP4 were not induced. Comparing the promoter regions upstream from the *GUS* gene in the constructs OP2 and OP3, OP2 had 562 bp region [−1451 bp to −889 bp relative to the transcriptional start site (TSS)] that was deleted from OP3. And the 562 bp fragment contains some stress-responsive cis-acting elements, such as W-box, ARR1AT, MBS. So we speculated that the 562 bp fragment was the key sequence involved in herbivore stress response. While the 290 bp fragment (−380 bp to −90 bp) was involved in herbivore stress response, this 562 bp fragment was then used for further research in this paper.

### Proteins bound to the herbivores-responsive promoter region

To determine the proteins bound to the herbivores-responsive promoter region, we used the 562 bp fragment mentioned above as a bait to screen transformants from a cDNA library generated from the leaves of rice plants infested with BPH for 24 h by yeast one-hybrid (Y1H) screening system. Through initial screening, DNA sequencing and BLAST analysis, some proteins including functional proteins and regulatory proteins showing interaction with *OsXIP* promoter were obtained. The functional proteins included fructose 1, 6-bisphosphatase, HSP, photosystem, Tify domain containing protein. While only two genes LOC_Os02g02480 and LOC_Os06g09390, according to the rice genome annotation of The Institute for Genomic Research (TIGR; http://rice.plantbiology.msu.edu/), were the candidates of regulatory proteins. Based on BLAST analysis and literatures, LOC_Os02g02480 is a basic helix-loop-helix protein (OsbHLH59) [11]; LOC_Os06g09390 is an APETALA2/ETHYLENE RESPONSE FACTOR (AP2/ERF) transcription factor (OsERF71) [12].

**Fig. 4:**
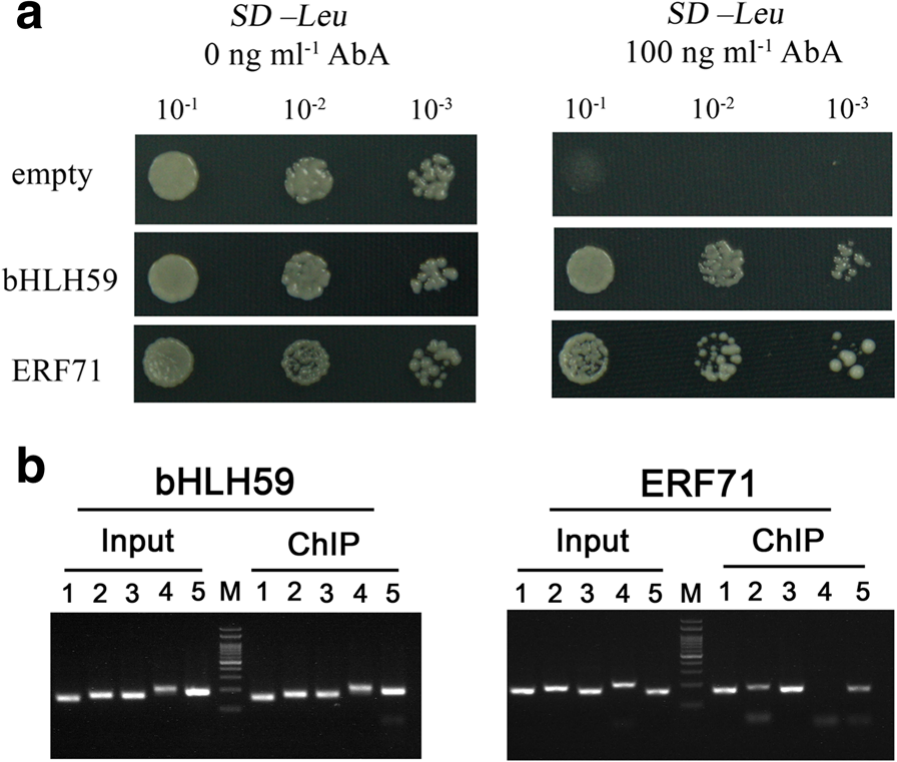
OsbHLH59 and OsERF71 bind to *OsXIP* promoter in vitro and in vivo. **a** TFs (OsbHLH59, and OsERF71) bind to *OsXIP* promoter in yeast. Bait strain Y1HGold[pBait-AbAi] yeast cells was transformed with a prey vector, containing OsbHLH59 and OsERF71 fused to a GAL4 activation domain, respectively. Cells were grown in liquid media to OD_600_ of 0.1 (10^−1^) and diluted in a 10× dilution series (10^−2^ to 10^−3^). Of each dilution, 7 μl was spotted on media selecting for both plasmids (SD/−Leu) and selecting for interaction (SD/−Leu/AbA^100^), supplemented with 100 ng ml^−1^ AbA to suppress background growth. **b** ChIP-PCR analysis. The ChIP of TFs (OsbHLH59, and OsERF71) assays was performed using transgenic rice expressing the 35S:OsbHLH59-GFP fusion or 35S:OsERF71-GFP fusion. Products of ChIP assays were amplified using five specific primers (listed in Additional file 1: Table S1)

The interaction between herbivores-responsive promoter region and the corresponding complete encoding products of the two genes were re-tested by Y1H assay. The analysis showed that the two proteins interacted specially with the promoter region (Fig. 4a). Meanwhile, we performed ChIP-PCR analysis by transforming 35S:OsbHLH59-sGFP and 35S:OsERF71-sGFP into rice to determine whether OsbHLH59 and OsERF71 regulate gene expression by binding to the 562 bp region in vivo. Our results showed that the promoter fragments of OsXIP were detected in the ChIP assays (Fig. 4b), further confirming that OsbHLH59 and OsERF71 can directly bind to the promoter motifs in vivo.

### Transcriptional activation of the *OsXIP* promoter by OsbHLH59 and OsERF71

**Fig. 5:**
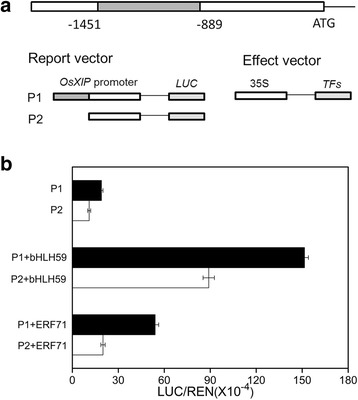
Tobacco transient transactivation assay for the interaction between OsbHLH59, OsERF71 and *OsXIP* promoter. **a** Characterization of *OsXIP* promoter and structures of vectors. Full length of the promoter from the translational start site (ATG) was indicated. OP2 and OP3 promoters were constructed into the report vector, and TFs (OsbHLH59, and OsERF71) were cloned into the effect vector, respectively. **b** Transient expression assay in *N. benthamiana*. LUC, Firefly luciferase activity; REN, Renilla luciferase activity (used as control). Data show ratios of LUC to REN and represent means ± SD of three independent replicates

We also performed the tobacco transient expression assay to further clarify the DNA-binding activities of the two proteins. The dual luciferases vector was used as a reporter system following Hellens et al. [22]. The full length ORFs of OsbHLH59 and OsERF71 were amplified and cloned into the effector vector. The OP2 and OP3 promoters were constructed into the reporter vector as P1 and P2 (Fig. 5a), which infiltrated *N. benthamiana* leaves alone or co-expressed with the corresponding effector vectors, respectively. It was obvious that co-expression of OsbHLH59 remarkably increased LUC expression driven by the OP2 promoter, as did expression of OsERF71 (Fig. 5b), suggesting OsbHLH59 and OsERF71 proteins promote the transcription of *OsXIP*. These results indicated that the two proteins may function as positive transcriptional regulators of *OsXIP* expression.

### Subcellular localization of OsbHLH59 and OsERF71

**Fig. 6:**
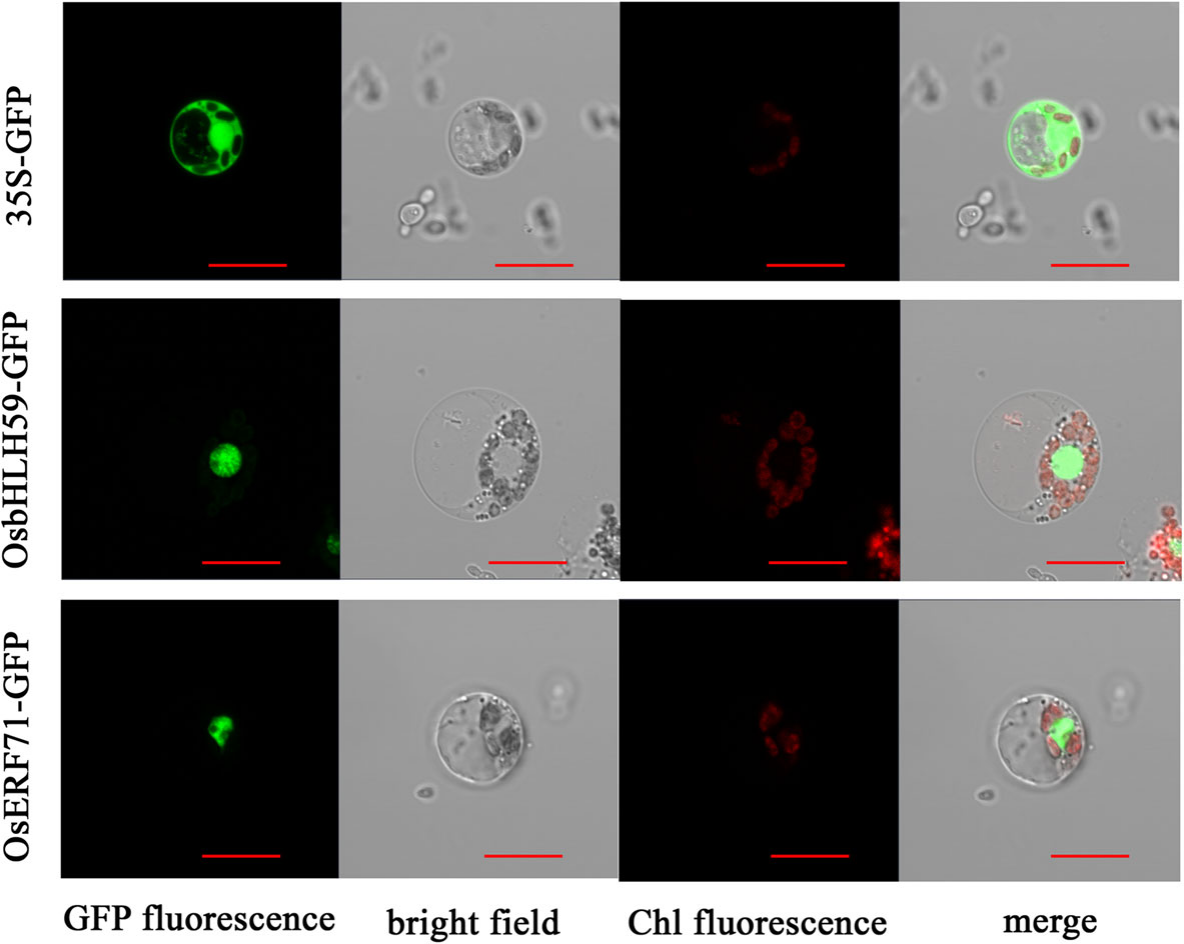
Subcellular localization of OsbHLH59 and OsERF71 in rice protoplasts. The rice protoplasts were transformed with 35Sp::OsbHLH59:GFP, 35Sp::OsERF71:GFP or pCAMBIA1300-GFP. The transformed cells were observed under a confocal microscope. The photographs were taken under detecting GFP fluorescence, bright field, chloroplast auto-fluorescence, and merged microscope images, respectively. Empty vector (pCAMBIA1300-GFP) was used as a control. *Bars*, 10 μm

To further evaluate the role of the OsbHLH59 and OsERF71 proteins, their subcellular localization were determined. We constructed 35Sp::OsbHLH59:GFP and 35Sp::OsERF71:GFP fusion genes, and transiently expressed the constructs in *N. benthamiana* leaves (Additional file 5: Figure S2) and rice protoplasts (Fig. 6), respectively. Fluorescence analysis revealed that the proteins localized only in the nucleus (Fig. 6). Cells infiltrated with GFP construct (control) yielded fluorescence both in the cytosol and the nucleus. These results further indicate that the two proteins may be transcriptional factors that function in the nucleus to regulate *OsXIP* expression.

### Influence of abiotic stress on the expression of transcription factors

**Fig. 7:**
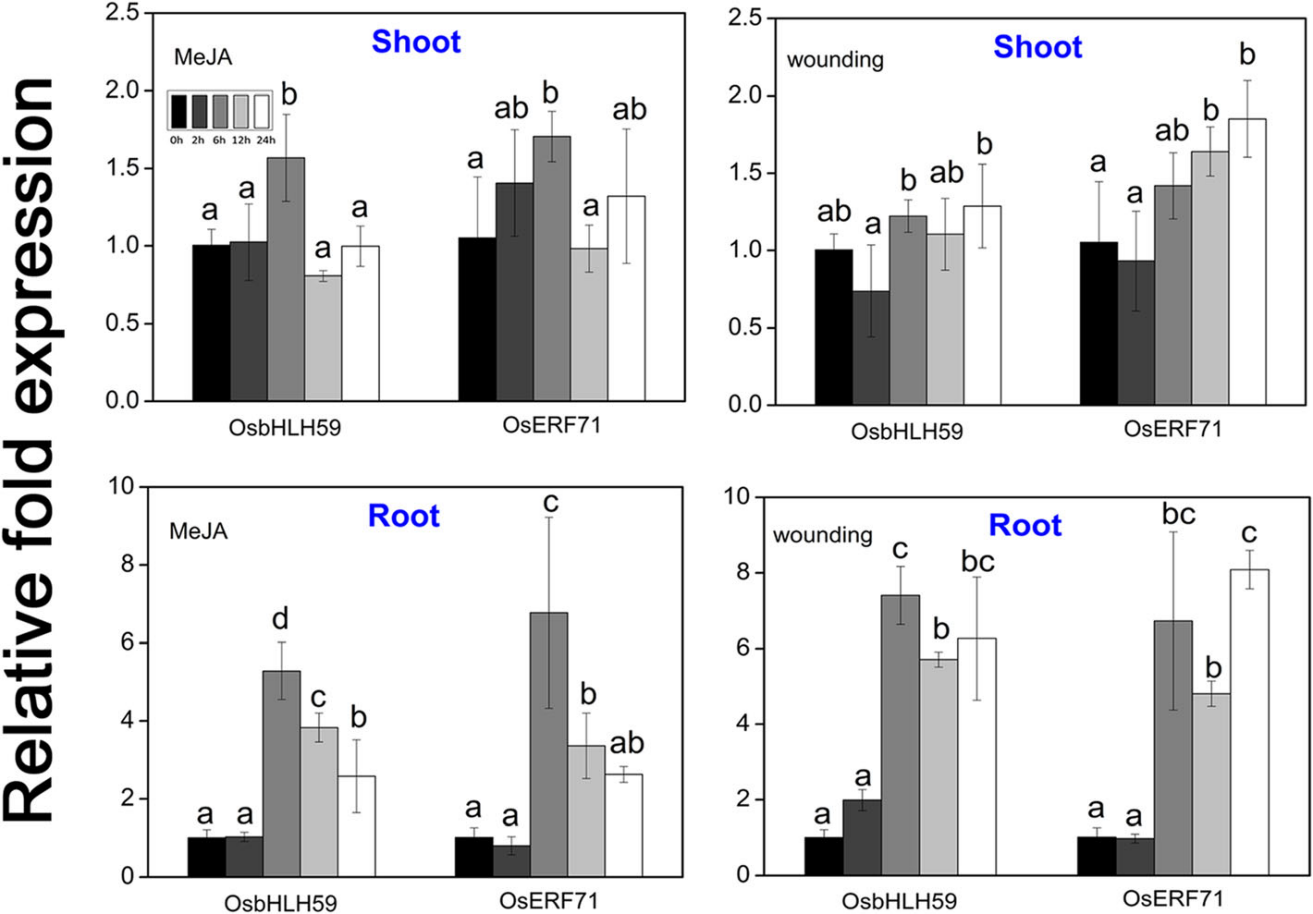
Expression patterns of the genes encoding transcription factors under abiotic stress analysed by qRT-PCR. Two-weeks-old WT seedlings were treated with 200 μM MeJA or wounding. Samples were collected immediately after treatment (0 h) and at 2 to 24 h after treatment. The data represent means ± SD of three independent replicates. Letters indicate significant differences between means using Duncan’s multiple range mean comparisons (5% α)

The expression patterns of the two genes that encode transcription factors bound to the rice herbivore-responsive cis-elements were examined by qRT-PCR in wild-type rice plants treated with MeJA or wounding at varying time intervals (2–24 h). The expression of these genes appeared to be responsive upon abiotic stress and was similar (Fig. 7). In shoot tissues, transcript expression of *OsbHLH59* and *OsERF71* increased concomitantly with time under MeJA treatment to 6 h, while decreased at 2 h and thereafter increased under wounding stress. The expression levels of *OsbHLH59* and *OsERF71* in root tissues were maximally induced approximately six- and seven-fold, and seven- and eight-fold after MeJA and wounding treatment, respectively. These results further suggest that the genes encoding these transcription factors may be involved in defence responses against herbivores by a JA- mediated pathway.

## Discussion

The phytohormone jasmonic acid (JA) play a vital role in plant defense when plants are exposed to invaders [26]. In general, herbivorous insects and necrotrophic pathogens are more sensitive to JA-induced defenses [27, 28]. Wounding that caused by mechanical injury or insect feeding, leads to the accumulation of JA. Subsequently, the JA pathway is activated, which induces JA-responsive gene expression [29]. Our results revealed that herbivore infestation, mechanical wounding and MeJA treatment enhanced the expression of OsXIP at the transcriptional and protein levels (Fig. 1), which is consistent with OsHI-XIP [5]. To date, there is one report that the xylanase inhibitor XIP-I could inhibit a xylanase from the digestive tract of the coffee berry borer [30]. *OsXIP* is a wound stress-responsive gene in rice [31]. When the herbivore feeds on the plants, wounding generates and then induces the accumulation of jasmonic acid (JA), which in turn activates wounding-associated defense pathways [32]. In our study, the expression level of OsXIP was up-regulated by BPH and the *N. lugens* induction process was very similar to that caused by wounding. When the rice was infested by BPH, wounding generated first and then activated the wound-responsive gene *OsXIP*, thus enhancing resistance against herbivores.

Bioinformatic analysis of the promoter of *OsXIP* revealed that it was a stress-induced promoter. *OsXIP* promoter contained stress-responsive cis-acting elements, such as ARR1AT, W-box, G-box and TGACG-motif. The W-box (TGACY), has been confirmed to be present upstream of salicylic acid or wound signal responsive genes; The G-box (CACGTG) and G-box-like (CANNTG, also called the E-box) are known to the binding sites of basic helix-loop-helix (bHLH) transcription factors [33]; TGACG motif has been reported to be essential for responsiveness to MeJA; ARR1AT and WRKY71OS exist in the promoter of growth regulators responsive genes. Thus, *OsXIP* gene responded to different stress (Fig. 1).

*OsXIP* promoter deletion analyses showed that GUS expression of OP3 reduced sharply compared with OP2, while OP4 slightly enhanced and OP5 reached a maximum (Fig. 2). This suggested there were at least an enhancer (*e*) and a repressor (*r*) motif between OP2 and OP3 (−1451 bp to −889 bp) and OP4 and OP5 (−569 bp to −380 bp), respectively. So a regulation model of the *OsXIP* gene was proposed: an enhancer site (*e*) may exist in −1451 bp to −889 bp, and a repressor site (*r*) may be located between −569 bp and −380 bp in the promoter. GUS expression in OP2 was high due to the binding of enhancer (E) to its site (*e*). The enhancer might preferentially bound to the promoter and facilitate the gene expression on the grounds that binding of enhancer E at *e* site prevents the repressor R from binding to *r* site by either binding with R or obstructing *r* site. GUS expression of OP3 and OP4 decreased because of deletion of enhancer site (*e*). GUS expression of OP5 and OP6 increased after deletion of the *r* site. Serial deletion promoter constructs between −1451 and −889 bp and between −569 and −380 bp can be constructed to determine the locations and sequences of *e* and *r* by transient expression assay and electrophoretic mobility shift assay (EMSA).

After BPH stress, a 562 bp region (−1451 to −889) in the *OsXIP* promoter were finally identified as the key sequences involved in the herbivores stress response (Fig. 3). By Y1H screening, positive DNA-protein binding in vitro and in vivo (Fig. 4) and nuclear localization (Fig. 6), OsbHLH59 and OsERF71 proteins were confirmed as the direct regulators of *OsXIP* expression. The two proteins activated the expression of *OsXIP* gene by transient transactivation assay (Fig. 5). Moreover, the two genes encoding corresponding OsbHLH59 and OsERF71 proteins showed differential expression upon MeJA and wounding stress (Fig. 7), further suggesting that they may be involved in positively regulating *OsXIP* expression via JA-mediated defence responses.

In the present study, two proteins (OsbHLH59, and OsERF71) as transcription activators of *OsXIP* were found. OsbHLH59 is a member of bHLH transcription factors, which are known to bind to G-box or E-box [11]. We analyzed the bait sequence and found that several E-box elements were present, including CAGTTG, CACTTG and CAATTG. And whether OsbHLH59 binds to these E-box elements of the bait can be further determined by EMSA. Some bHLH transcription factors in rice are involved in stress responses. For instance, RERJ1 (OsbHLH006) responded to wound and drought [34, 35]; OsBP-5 (OsbHLH102) is related to transcriptional regulation of the rice *Wx* gene [36]; OsbHLH094 forms a complex with RSS3 and JASMONATE ZIM-DOMAIN (JAZ) proteins to modulate the expression of JA-responsive genes [37]; DPF (OsbHLH25) positively regulates the accumulation of diterpenoid phytoalexins [38].

In *Arabidopsis*, the bHLH transcription factor MYC2 is a key positive transcriptional regulator of JA signaling pathway, which is inhibited by JAZ transcriptional repressors [39, 40]. Similarly, OsbHLH062 interacted with OsJAZ9 to regulate JA-responsive genes expression in rice [41]. Furthermore, the JA signaling pathway in *Arabidopsis* composes of the two major branches: the ERF branch and MYC branch [29]. And the ERF branch is controlled by AP2/ERF transcription factors, such as ORA59 [42]. Thus, the OsERF71 protein, an AP2/ERF transcription factor, may belong to the ERF branch to positively regulate the expression of *OsXIP*. In addition, transcriptional analysis revealed mechanical wounding and MeJA induced transcriptional expression of *OsbHLH59* and *OsERF71* in rice (Fig. 7). These results reinforce the possibility that the induction expression of *OsXIP* by wounding may be regulated by the JA-mediated signaling pathway [2]. So OsbHLH59 and OsERF71 may belong to the MYC branch and the ERF branch, respectively, to positively regulate the expression of *OsXIP*. This speculation is further supported by the findings that *OsXIP* was independent of growth and development in rice plants [30] and its expression was not regulated by phytohormones associated with growth [2].

## Conclusions

In summary, we reveal the transcriptional regulatory mechanism of OsXIP and its involvement in defense response in rice. In response to herbivore infestation, mechanical wounding or MeJA stress, OsbHLH59 and OsERF71 transcription factors promote this process by activating the expression of *OsXIP* via directly binding to its promoter. Our discovery contributes to clarify the regulatory mechanism of OsXIP and gives us a better understanding of the function of OsXIP in plant defence.

## Additional files

Additional file 1: Table S1. List of primers used for gene constructs. (DOCX 18 kb)
Additional file 2: Table S2. Primers for quantitative real-time (qRT) PCR. (DOCX 14 kb)
Additional file 3: Table S3. Primers for chromatin immunoprecipitation (ChIP)-PCR. (DOCX 19 kb)
Additional file 4: Figure S1. The sequence of the putative promoter region (−2,070/+52) of *OsXIP*. The transcription start site is indicated as +1, and the putative start codon is underlined; All potential cis-acting elements are boxed; All the primers (forward OP1-U- OP7-U and reverse OP-L) are indicated by red arrows. (DOCX 793 kb)
Additional file 5: Figure S2. Subcellular localization of OsbHLH59 and OsERF71 in *N. benthamiana*. The full length ORFs without terminators of OsbHLH59 and OsERF71 were cloned into the pCAMBIA1300-sGFP vector under the control of the 35S promoter. Then *N. benthamiana* cells were transformed with 35Sp::OsbHLH59:GFP, 35Sp::OsERF71:GFP or pCAMBIA1300-GFP. After incubating for 48 h, the transformed cells were observed under a confocal microscope. The photographs were taken under detecting GFP fluorescence, bright field, and in combination (merge), respectively. Empty vector (pCAMBIA1300-GFP) was used as a control. *Bars*, 10 μm. (DOCX 1068 kb)

## Abbreviations

BPH: Brown planthopper
ChIP: Chromatin immunoprecipitation
GFP: Green fluorescent protein
GUS: β-glucuronidase
JA: Jasmonic acid
JAZ: JASMONATE ZIM-DOMAIN
MeJA: Methyl jasmonate
XIs: Xylanase inhibitors
