# Supplementary material for: Identification of two transcription factors activating the expression of OsXIP in rice defence response
Source: BMC Biotechnol. 2017 Mar 7;17:26. doi: 10.1186/s12896-017-0344-7 (PMC5341196; doi:10.1186/s12896-017-0344-7)
Supplement: Additional file 1: Table S1. — List of primers used for gene constructs. (DOCX 18 kb) [file 12896_2017_344_MOESM1_ESM.docx]

**Table S1.** List of primers used for gene constructs

| Primer name | Primers sequence (from 5’ to 3’) | Restriction enzymes |
| --- | --- | --- |
| OP1-U | CCCAAGCTTAGCTCCCCCTTGATCAATTGC | *Hind* III |
| OP1-L | CGGATCCTGTTGATATGTTGGTCGAATCG | *BamH* I |
| OP2-U | CCCAAGCTTTGTCATCTCGGTGCAGTTGTG | *Hind* III |
| OP3-U | CCCAAGCTTGCACCGTAGGCCCAATCCAC | *Hind* III |
| OP4-U | CCCAAGCTTCCTGTGACCACTGAAATTGCC | *Hind* III |
| OP5-U | CCCAAGCTTGTAATTGGTGGCGTGCTGGG | *Hind* III |
| OP6-U | CCCAAGCTTTGGTGGTGAAGCGTGAAGG | *Hind* III |
| OP7-U | CCCAAGCTTGCGCGCACACAACATAAGAC | *Hind* III |
| 59-AD-U  59-AD-L  71-AD-U  71-AD-L | GAATTCATGGACGGAGGCGGAGACCCC  GGATCCTTAAGCATTTGGTGGCCAAAG  GAATTCATGTGCGGCGGCGCCATCCT  GGATCCTCAGTAGAACTCGGCCGACAC | *EcoR* I  *BamH* I  *EcoR* I  *BamH* I |
| 59-GFP-U  59-GFP-L  71-GFP-U  71-GFP-L | GAGCTCATGGACGGAGGCGGAGACCCC  TCTAGAAGCATTTGGTGGCCAAAG  GGATCCATGTGCGGCGGCGCCATCCT  GTCGACGTAGAACTCGGCCGACAC | *Sac* I  *Xba* I  *BamH* I  *Sal* I |
