# Supplementary material for: Identification of two transcription factors activating the expression of OsXIP in rice defence response
Source: BMC Biotechnol. 2017 Mar 7;17:26. doi: 10.1186/s12896-017-0344-7 (PMC5341196; doi:10.1186/s12896-017-0344-7)
Supplement: Additional file 2: Table S2. — Primers for quantitative real-time (qRT) PCR. (DOCX 14 kb) [file 12896_2017_344_MOESM2_ESM.docx]

**Table S2.** Primers for quantitative real-time (qRT) PCR

| Name | Gene ID | Primers sequence (from 5’ to 3’) |
| --- | --- | --- |
| *Osactin* | Os03g50885 | TCAGCAACTGGGATGATATGGAG GCCGTTGTGGTGAATGAGTAAC |
| *OsXIP* | AK073843 | CAACAAGGACTACCGCGCCAC  AAACCATGACGCCTCCGAAGT |
| *OsbHLH59* | LOC_Os02g02480 | GCAGAGAAGGAGAGGCTGGA  ACCACTGCCACATTGCCATC |
| *OsERF71* | LOC_Os06g09390 | GGCCGTCATGTCAGCAGG  GGTCGGTGCAAGCATGGA |
