# Supplementary material for: Identification of two transcription factors activating the expression of OsXIP in rice defence response
Source: BMC Biotechnol. 2017 Mar 7;17:26. doi: 10.1186/s12896-017-0344-7 (PMC5341196; doi:10.1186/s12896-017-0344-7)
Supplement: Additional file 3: Table S3. — Primers for chromatin immunoprecipitation (ChIP)-PCR. (DOCX 19 kb) [file 12896_2017_344_MOESM3_ESM.docx]

**Table S3.** Primers for Chromatin immunoprecipitation (ChIP)-PCR

| Name | Gene ID | Number | Primers sequence (from 5’ to 3’) |
| --- | --- | --- | --- |
| *OsXIP*  (OsbHLH59) | AK073843 | 1 | TGATGTTGGTGCTTACTAGG |
|  |  |  | AACAATTACATCATGGGTCA |
|  |  | 2 | CTTAACGGTCAGTTGTTGTG |
|  |  |  | AGGAGTCCAAACTTAAGTAAA |
|  |  | 3 | GATGACCCATGATGTAATTG |
|  |  |  | TGACCCCAAATCGTATATGT |
|  |  | 4 | ACGGATTACCATTGGATACA |
|  |  |  | AACCCAGCAAGTGTGTCTCT |
|  |  | 5 | TGTCATCTCGGTGCAGTTGT |
|  |  |  | AAAAAGAAGTCACACAAGCG |
| *OsXIP*  (OsERF71) | AK073843 | 1 | CTTAACGGTCAGTTGTTGTG |
|  |  |  | AGGAGTCCAAACTTAAGTAAA |
|  |  | 2 | CGGGTACTTCTATACAACAGG |
|  |  |  | ACAATTACATCATGGGTCATC |
|  |  | 3 | TAGAGATTGAGGAGGACCTG |
|  |  |  | AAGAGAGTAAAACACAGCGA |
|  |  | 4 | GCAGAGTTTAAATCTATTCGG |
|  |  |  | CCTGTTGTATAGAAGTACCCG |
|  |  | 5 | GATGACCCATGATGTAATTG |
|  |  |  | TGACCCCAAATCGTATATGT |
