# Supplementary material for: Identification of two transcription factors activating the expression of OsXIP in rice defence response
Source: BMC Biotechnol. 2017 Mar 7;17:26. doi: 10.1186/s12896-017-0344-7 (PMC5341196; doi:10.1186/s12896-017-0344-7)

**Additional file 4: Figure S1.** The sequence of the putative promoter region (-2,070/+52) of *OsXIP*. The transcription start site is indicated as +1, and the putative start codon is underlined; All potential cis-acting elements are boxed; All the primers (forward OP1-U- OP7-U and reverse OP-L) are indicated by red arrows.
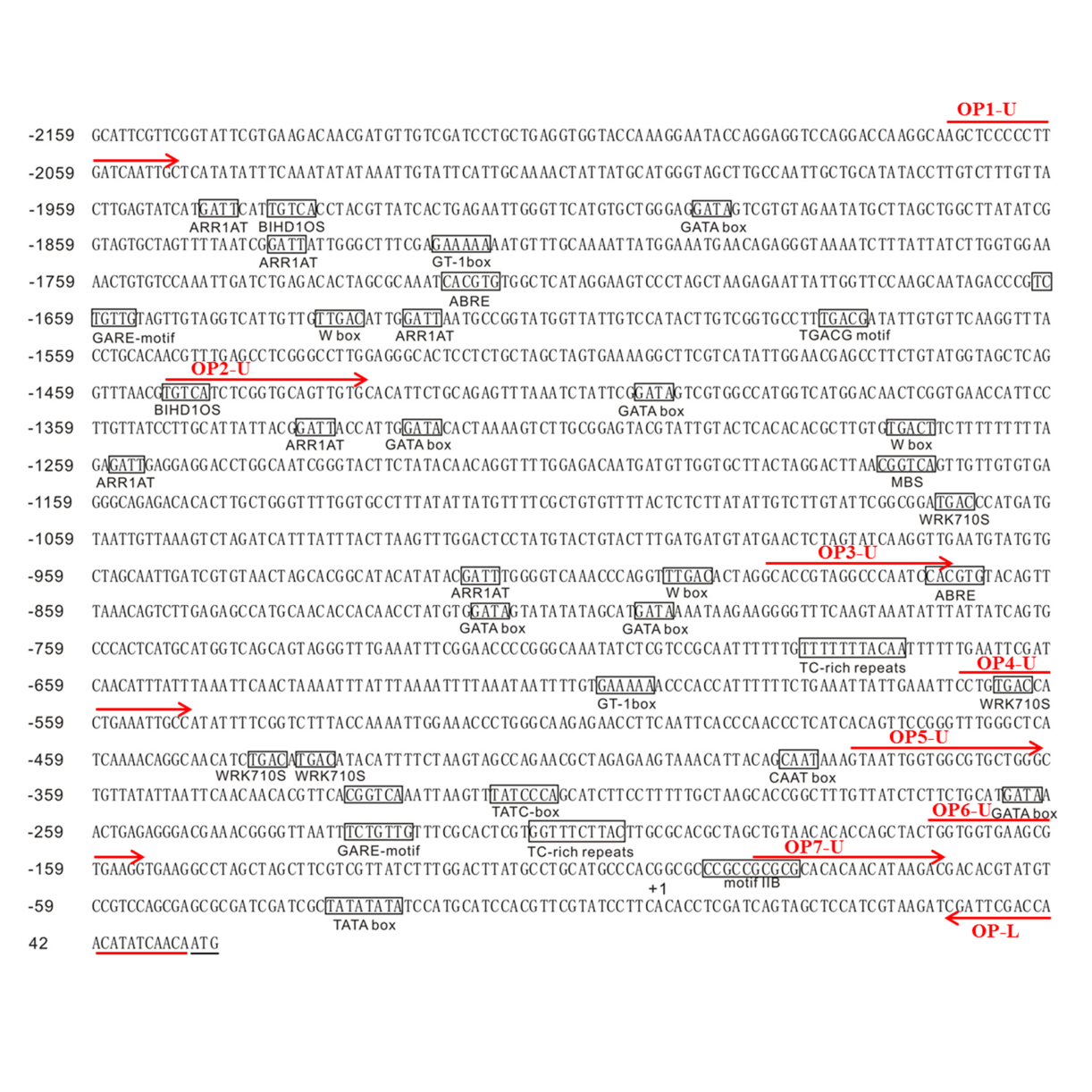

Supplement: Additional file 4: Figure S1. — The sequence of the putative promoter region (−2,070/+52) of OsXIP. The transcription start site is indicated as +1, and the putative start codon is underlined; All potential cis-acting elements are boxed; All the primers (forward OP1-U- OP7-U and reverse OP-L) are indicated by red arrows. (DOCX 793 kb) [file 12896_2017_344_MOESM4_ESM.docx]
