# Supplementary material for: Identification of two transcription factors activating the expression of OsXIP in rice defence response
Source: BMC Biotechnol. 2017 Mar 7;17:26. doi: 10.1186/s12896-017-0344-7 (PMC5341196; doi:10.1186/s12896-017-0344-7)

**Additional file 5: Figure S2.** Subcellular localization of OsbHLH59 and OsERF71 in *N. benthamiana*. The full length ORFs without terminators of OsbHLH59 and OsERF71 were cloned into the pCAMBIA1300-sGFP vector under the control of the 35S promoter. Then *N. benthamiana* cells were transformed with 35Sp::OsbHLH59:GFP, 35Sp::OsERF71:GFP or pCAMBIA1300-GFP. After incubating for 48 h, the transformed cells were observed under a confocal microscope. The photographs were taken under detecting GFP fluorescence, bright field, and in combination (merge), respectively. Empty vector (pCAMBIA1300-GFP) was used as a control. *Bars*, 10 μm


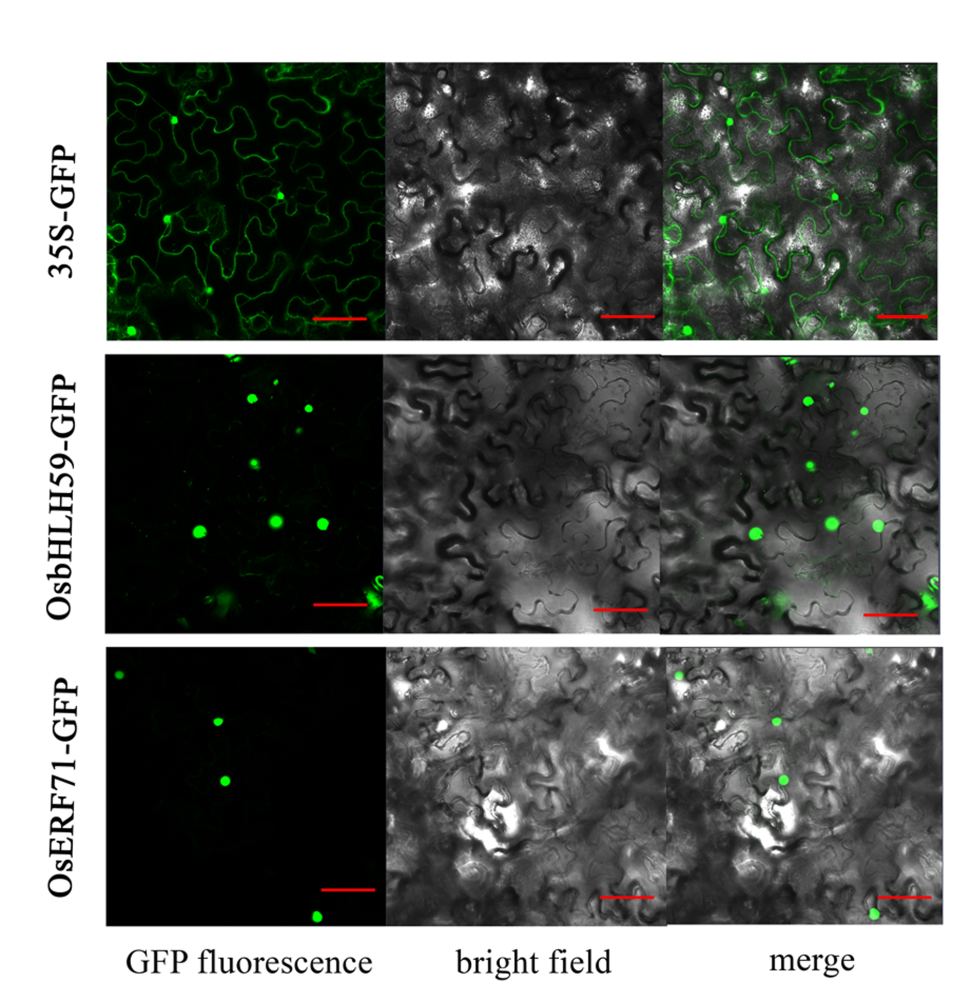

Supplement: Additional file 5: Figure S2. — Subcellular localization of OsbHLH59 and OsERF71 in N. benthamiana. The full length ORFs without terminators of OsbHLH59 and OsERF71 were cloned into the pCAMBIA1300-sGFP vector under the control of the 35S promoter. Then N. benthamiana cells were transformed with 35Sp::OsbHLH59:GFP, 35Sp::OsERF71:GFP or pCAMBIA1300-GFP. After incubating for 48 h, the transformed cells were observed under a confocal microscope. The photographs were taken under detecting GFP fluorescence, bright field, and in combination (merge), respectively. Empty vector (pCAMBIA1300-GFP) was used as a control. Bars, 10 μm. (DOCX 1068 kb) [file 12896_2017_344_MOESM5_ESM.docx]
